# Supplementary material for: D-(+)-Galactose-induced aging: A novel experimental model of erectile dysfunction
Source: PLoS One. 2021 Apr 15;16(4):e0249487. doi: 10.1371/journal.pone.0249487 (PMC8049229; doi:10.1371/journal.pone.0249487)
Supplement: Attachment — Submitted filename: comments.docx [file pone.0249487.s002.docx]

Comments

1. There are minor typing errors:

- Line 29- DE replace to ED
- Line 348-the activity replace to The activity

2. In the material-method section, it should be stated how the D- (g+) galactose and sildenafil doses are selected. Because, as it is known, the dose of the research molecule is the main parameter in studies investigating molecular mechanisms.

3. In the result part, why were DGAL + SD1.5 results not presented in the relaxation study results (between the 232-235^th^ sentences)?

4. In the discussion section, the reason for the improvement in ICP / MAP in the DGAL + SD1.5 group was discussed as cGMP increase mediated by PDE-5 inhibition by sildenafil (324-329^th^ sentences). However, recent studies have shown that sildenafil increases NO bioavailability by reducing oxidative stress and restoring pro-oxidant / antioxidant balance (Leal MAS et al. Sildenafil reduces aortic endothelial dysfunction and structural damage in spontaneously hypertensive rats: Role of NO, NADPH and COX-1 pathways. Vascul Pharmacol 124:106601; 2020). Considering the possible mechanism of D-galactose in aging "oxidation to form hydrogen peroxide by galactose oxidase, ROS increase resulting in decreased SOD level and impaired redox homeostasis", (Azman KF, Zakaria R. D-galactose-induced accelerated aging model: an overview. Biogerontology 20:763-782, 2019). I believe that discussing the effect of sildenafil mentioned above in this section will strengthen the results of the study.

5. In the discussion section, it was not understood what was meant by SHR-CTL and WKY-CTL groups between sentences 344-353. In the material method part, no information about these groups is given.

6. In the discussion section, between the 354-363^th^ sentences Could the decrease in acetylcholine response be due to a decrease in NO bioavailability rather than a decrease in NO release in DGAL group? The results in the DGAL + SD1.5 group specified in Article 3 can be discussed considering. Because as it is known, in the presence of ROS, ONOO^-^ produced by interacting with NO and ROS. This molecule is the main molecule responsible for nitrosative damage and reduced NO bioavailability. Again, as discussed in Article 4; it was shown that, Sildenafil reverses endothelial dysfunction in spontaneous hypertensive rats by improving vascular relaxation to acetylcholine with increased NO bioavailability via reducing oxidative stress (Azman KF, Zakaria R. D-galactose-induced accelerated aging model: an overview. Biogerontology 20:763-782, 2019).

7. In the discussion section, in the paragraph starting with the 385th sentence, morphological changes are mentioned, and a decrease in smooth muscle cells in the DGAL group is stated. Does this not contradict the nitroprusside non-response referred to in sentences 364-369? Researchers should discuss this and, if necessary, state that the morphological changes are not at a level to create functional changes.
