# Supplementary material for: D-(+)-Galactose-induced aging: A novel experimental model of erectile dysfunction
Source: PLoS One. 2021 Apr 15;16(4):e0249487. doi: 10.1371/journal.pone.0249487 (PMC8049229; doi:10.1371/journal.pone.0249487)
Supplement: Attachment — Submitted filename: Response to the editor final.docx [file pone.0249487.s003.docx]

**Response to the editor**

1. **Please ensure that your manuscript meets PLOS ONE's style requirements, including those for file naming.**

Answer: The PLOS ONE’s style requirements have been met.

2. **Please note that PLOS does not permit references to “data not shown.” Authors should provide the relevant data within the manuscript, the Supporting Information files, or in a public repository. If the data are not a core part of the research study being presented, we ask that authors remove any references to these data.**

Answer: References to "data not shown" have been removed. However, we have performed a new set of experiments to follow reviewer’s suggestions, where we have constructed concentration-response curves to SNP in DGAL treated rats. These new data were presented and discussed in the revised the version of manuscript.

3. **Please ensure you have discussed any potential limitations of your study in the Discussion.**

Answer: As we know, D-gal is a normal substance in the body, however, at high levels, accumulating free D-gal is converted into secondary metabolites such as galactitol, hydrogen peroxide, Schiff’s base, that, in turn, induce inflammation, cellular apoptosis, degenerative changes, resulting in aging and age-related disorders. Furthermore, this model is characterized by increased inflammatory cytokines, up-regulated P16, P53 and P21 genes expressions [13, 26, 50]. So that one of the main limitations of the present study is that this model can be hard to reflect real physiology and biochemistry changes. In addition, we have not measured inflammatory mediators, P53-P21, PI3K/Akt and AMPK/ ULK1 pathways in the present study. Regardless, D-galactose- induced aging carry the potential to be an ideal model for anti-aging therapeutic interventions studies due to its abilities to mimic senescence characteristics of natural aging. This paragraph has been added to the discussion section (see sentences 438-449).

4. **At this time, we request that you please report additional details in your Methods section regarding animal care, as per our editorial guidelines:**
(1) **Please state the number of rats used in the study**

Answer: We added the information concerning the number of animals used in the study (see sentence 85).

“Forty male Wistar rats (Rattus novergicus), eight weeks old, from the Animal Production Unit of the Institute for Research in Drugs and Medicines (IPeFarM) of the Federal University of Paraíba (UFPB) were used.”

(2) **Please include the method of euthanasia. Please also clarify whether animals were euthanised prior to isolation of the penis.**

Answer: The euthanasia method was added to the manuscript (sentence 90-93), as well as if the animals were euthanized before the isolation of the corpus cavernosum (sentence 141).

Sentence 90-93: “The animals were euthanized by exsanguination, after confirmation of anesthesia induced by the intraperitoneally administration of xylazine and ketamine (10 and 75 mg/Kg, respectively).”

Sentence 141: “After euthanasia, the animal's penises was carefully isolated and immediately placed in a Krebs-Ringer nutrient solution with composition (mM): NaCl 118.0; KCl 4.7; CaCl_2_ 2.50; KH_2_PO_4_ 1.20; MgSO_4_ 1.17; NaHCO_3_ 25.00 and glucose 5.60, for dissection and removal of the corpus cavernosum (21).”

(3) **Please describe the care received by the animals, including the frequency of monitoring and the criteria used to assess animal health and well-being.**

Answer: Animal care was described, as well as the frequency of monitoring and the criteria used to assess the animal's health and well-being (see sentence 87-90 and 94-98).

Sentence 87-90: “The animals were kept under appropriate environmental conditions, temperature (22 ± 1 ° C), 12-hour light-dark cycle (6-18 hours), with free access to water and food (Nuvilab CR-1, Quimtia®), keeping track of the physical and mental health of the animals on a daily basis.”

Sentence 94-98: “All experimental protocols were carried out according to the guidelines established by the brazilian National Council for Animal Experiment Control (Conselho Nacional de Controle de Experimentação Animal - CONCEA), obeying the law 11.794/2008, submitted and previously approved by the Ethics Committee on the Use of Animals (Comissão de Ética no Uso de Animais - CEUA) of the UFPB, nº 9706070319.”

5. **Please ensure you have correctly described the number of experimental groups in line 92.**

**Answer:** We appreciate the suggestions. Typing errors were corrected in the manuscript (see sentence 100).

“The animals were randomly assigned into three experimental groups: control group (CTL), which received physiological saline solution (NaCl 0.9%) intraperitoneally (IP), D-galactose group (DGAL), which received D-(+)-galactose 150 mg/Kg via IP and sildenafil group (DGAL+SD1.5) which received D-(+)-galactose 150 mg/Kg via IP and sildenafil 1.5 mg/Kg by oral gavage.”

6. **At this time, we ask that you please provide scale bars on the microscopy images presented in Figures 4 and 6, and refer to the scale bar in the corresponding Figure legend.**

Answer: The scale bars of the images were added, as requested, and the reference to it in the caption of the corresponding figure (see figure 4 and 6) (see sentence 278, 289 and 302).

Sentence 278: Scale bars, 100 μm.

Sentence 289: Scale bars, 200 μm.

Sentence 302: Scale bars, 100 μm.

7. **We note that you have indicated that data from this study are available upon request. PLOS only allows data to be available upon request if there are legal or ethical restrictions on sharing data publicly. For more information on unacceptable data access restrictions, please see http://journals.plos.org/plosone/s/data-availability#loc-unacceptable-data-access-restrictions.**

Answer: We have mentioned (see sentences 94-98) that all experimental protocols were carried out according to the guidelines established by the brazilian National Council for Animal Experiment Control (Conselho Nacional de Controle de Experimentação Animal - CONCEA), obeying the law 11.794/2008, submitted and previously approved by the Ethics Committee on the Use of Animals (Comissão de Ética no Uso de Animais - CEUA) of the Federal University of Paraiba-UFPB, nº 9706070319.

Importantly, none of the funding institutions restricts the availability of data. All data will be fully available. A file containing all results spreadsheets, was submitted along with manuscript.
