# Supplementary material for: D-(+)-Galactose-induced aging: A novel experimental model of erectile dysfunction
Source: PLoS One. 2021 Apr 15;16(4):e0249487. doi: 10.1371/journal.pone.0249487 (PMC8049229; doi:10.1371/journal.pone.0249487)
Supplement: Attachment — Submitted filename: Response to the editor final.docx [file pone.0249487.s004.docx]

**Response to the editor**

1. **Please ensure that your manuscript meets PLOS ONE's style requirements, including those for file naming.**

Answer: The PLOS ONE’s style requirements have been met.

2. **Please note that PLOS does not permit references to “data not shown.” Authors should provide the relevant data within the manuscript, the Supporting Information files, or in a public repository. If the data are not a core part of the research study being presented, we ask that authors remove any references to these data.**

Answer: References to "data not shown" have been removed. However, we have performed a new set of experiments to follow reviewer’s suggestions, where we have constructed concentration-response curves to SNP in DGAL treated rats. These new data were presented and discussed in the revised the version of manuscript.

3. **Please ensure you have discussed any potential limitations of your study in the Discussion.**

Answer: D-gal is a known normal substance in the body, however, at high levels, accumulating free D-gal is converted into secondary metabolites such as galactitol, hydrogen peroxide, and Schiff’s base, which in turn, induce inflammation, cellular apoptosis, and degenerative changes, this resulting in aging and age-related disorders. Further, this model was characterized by increased inflammatory cytokines, and up-regulated P16, P53, and P21 gene expression [13, 26, 50]. One of the main limitations of the present study is that the model poorly relates real physiological and biochemical changes. In addition, in the present study, inflammatory mediators, P53-P21, PI3K/Akt, and AMPK/ULK1 pathways were not measured. Nevertheless, due to its ability to mimic the senescent characteristics of natural aging, D-galactose-induced aging is potentially an ideal model for anti-aging therapeutic intervention studies. This paragraph has been added to the discussion section (see sentences 421-431).

4. **At this time, we request that you please report additional details in your Methods section regarding animal care, as per our editorial guidelines:**
(1) **Please state the number of rats used in the study**

Answer: We added the information concerning the number of animals used in the study (see sentence 84-86).

“Forty male Wistar rats (Rattus novergicus), eight weeks old, from the Animal Production Unit of the Institute for Research in Drugs and Medicines (IPeFarM) of the Federal University of Paraíba (UFPB) were used.”

(2) **Please include the method of euthanasia. Please also clarify whether animals were euthanised prior to isolation of the penis.**

Answer: The euthanasia method was added to the manuscript (sentence 89-92). Furthermore, the animals were euthanized before the isolation of the corpus cavernosum (sentence 137).

Sentence 89-92: “After confirmation of anesthesia induced by the intraperitoneally administration of xylazine and ketamine (10 and 75 mg/Kg, respectively), the animals were euthanized by exsanguination”

Sentence 137: “After euthanasia, the animal's penises were carefully isolated and immediately placed in a Krebs-Ringer nutrient solution (NaCl 118.0; KCl 4.7; CaCl_2_ 2.50; KH_2_PO_4_ 1.20; MgSO_4_ 1.17; NaHCO_3_ 25.00; and glucose 5.60 (mM)) for dissection and removal of the corpus cavernosum [21]”

(3) **Please describe the care received by the animals, including the frequency of monitoring and the criteria used to assess animal health and well-being.**

Answer: Animal care was described, as well as the frequency of monitoring and the criteria used to assess the animal's health and well-being (see sentence 86-89 and 93-97).

Sentence 86-89: “The animals were kept under appropriate environmental conditions, temperature (22 ± 1°C), a 12-hour light-dark cycle (6-18 hours), with free access to water and food (Nuvilab CR-1, Quimtia®), while recording the physical and mental health of the animals on a daily basis.”

Sentence 93-97: “All experimental protocols were carried out according to the guidelines established by the Brazilian National Council for Animal Experiment Control (Conselho Nacional de Controle de Experimentação Animal - CONCEA), obeying law No. 11.794/2008, submitted and previously approved by the Ethics Committee on the Use of Animals (Comissão de Ética no Uso de Animais - CEUA) of the UFPB, nº 9706070319.”

5. **Please ensure you have correctly described the number of experimental groups in line 92.**

**Answer:** We appreciate the suggestions. Typing errors were corrected in the manuscript (see sentence 99).

“The animals were randomly assigned into ~~two~~ three experimental groups: the control group (CTL), which received physiological saline solution (NaCl 0.9%) intraperitoneally (IP), the D-galactose group (DGAL), which received D-(+)-galactose at 150 mg/Kg via IP, and the sildenafil group (DGAL+SD1.5) which received both D-(+)-galactose at 150 mg/Kg via IP and sildenafil 1.5 mg/Kg by oral gavage.”

6. **At this time, we ask that you please provide scale bars on the microscopy images presented in Figures 4 and 6, and refer to the scale bar in the corresponding Figure legend.**

Answer: The scale bars of the images were added, as requested, and the reference to it in the caption of the corresponding figure (see figure 4 and 6) (see sentence 272, 282 and 294).

Sentence 272: Scale bars, 100 μm.

Sentence 282: Scale bars, 200 μm.

Sentence 294: Scale bars, 100 μm.

7. **We note that you have indicated that data from this study are available upon request. PLOS only allows data to be available upon request if there are legal or ethical restrictions on sharing data publicly. For more information on unacceptable data access restrictions, please see http://journals.plos.org/plosone/s/data-availability#loc-unacceptable-data-access-restrictions.**

Answer: We have mentioned (see sentences 93-97) that All experimental protocols were carried out according to the guidelines established by the Brazilian National Council for Animal Experiment Control (Conselho Nacional de Controle de Experimentação Animal - CONCEA), obeying law No. 11.794/2008, submitted and previously approved by the Ethics Committee on the Use of Animals (Comissão de Ética no Uso de Animais - CEUA) of the UFPB, nº 9706070319.

Importantly, none of the funding institutions restricts the availability of data. All data will be fully available. A file containing all results spreadsheets, was submitted along with manuscript.
